# Neuromedin B receptor stimulation of Cav3.2 T-type Ca^2+^ channels in primary sensory neurons mediates peripheral pain hypersensitivity

**DOI:** 10.7150/thno.62255

**Published:** 2021-09-09

**Authors:** Yuan Zhang, Zhiyuan Qian, Dongsheng Jiang, Yufang Sun, Shangshang Gao, Xinghong Jiang, Hua Wang, Jin Tao

**Affiliations:** 1 Department of Geriatrics & Institute of Neuroscience, the Second Affiliated Hospital of Soochow University, Suzhou 215004, China.; 2 Comprehensive Pneumology Center, Helmholtz Zentrum München, Munich 81377, Germany.; 3 Department of Physiology and Neurobiology & Centre for Ion Channelopathy, Medical College of Soochow University, Suzhou 215123, China.; 4 Department of Endocrinology, Shanghai East Hospital, Tongji University School of Medicine, Shanghai 200120, China.; 5 Jiangsu Key Laboratory of Neuropsychiatric Diseases, Soochow University, Suzhou 215123, China.

**Keywords:** T-type Ca^2+^ channel, neuromedin B receptor, trigeminal ganglion neurons, protein kinase A

## Abstract

**Background:** Neuromedin B (Nmb) is implicated in the regulation of nociception of sensory neurons. However, the underlying cellular and molecular mechanisms remain unknown.

**Methods:** Using patch clamp recording, western blot analysis, immunofluorescent labelling, enzyme-linked immunosorbent assays, adenovirus-mediated shRNA knockdown and animal behaviour tests, we studied the effects of Nmb on the sensory neuronal excitability and peripheral pain sensitivity mediated by Cav3.2 T-type channels.

**Results:** Nmb reversibly and concentration-dependently increased T-type channel currents (*I*_T_) in small-sized trigeminal ganglion (TG) neurons through the activation of neuromedin B receptor (NmbR). This NmbR-mediated *I*_T_ response was G_q_ protein-coupled, but independent of protein kinase C activity. Either intracellular application of the QEHA peptide or shRNA-mediated knockdown of G_β_ abolished the NmbR-induced *I*_T_ response. Inhibition of protein kinase A (PKA) or AMP-activated protein kinase (AMPK) completely abolished the Nmb-induced *I*_T_ response. Analysis of phospho-AMPK (*p*-AMPK) revealed that Nmb significantly activated AMPK, while AMPK inhibition prevented the Nmb-induced increase in PKA activity. In a heterologous expression system, activation of NmbR significantly enhanced the Cav3.2 channel currents, while the Cav3.1 and Cav3.3 channel currents remained unaffected. Nmb induced TG neuronal hyperexcitability and concomitantly induced mechanical and thermal hypersensitivity, both of which were attenuated by T-type channel blockade. Moreover, blockade of NmbR signalling prevented mechanical hypersensitivity in a mouse model of complete Freund's adjuvant-induced inflammatory pain, and this effect was attenuated by siRNA knockdown of Cav3.2.

**Conclusions:** Our study reveals a novel mechanism by which NmbR stimulates Cav3.2 channels through a G_βγ_-dependent AMPK/PKA pathway. In mouse models, this mechanism appears to drive the hyperexcitability of TG neurons and induce pain hypersensitivity.

## Introduction

Neuromedin B (Nmb) is a highly conserved and non-glycosylated polypeptide in mammals initially purified from porcine spinal cord that serves as a neuromodulator by paracrine or autocrine mechanisms 1. Nmb receptor (NmbR), which is located predominantly (but not exclusively) in peripheral tissues and organs and the central nervous system in mammals, has been identified as an endogenous receptor for Nmb 2. Acting through this receptor, Nmb mediates several behavioural effects and performs various biological functions, including regulating food intake, smooth muscle contraction, body temperature, and stress responses 3. Additionally, recent *in vivo* and *in vitro* studies have demonstrated the functional role of Nmb in nociception and have suggested that Nmb is a key determinant of nociceptive sensitivity in primary afferent neurons 3, 4. For instance, NmbR protein expression has been detected in sensory neurons, and behavioural analysis has shown that intrathecal injection of Nmb elicits nociceptive reflex responses 4, 5. Nevertheless, direct proof and the detailed molecular basis of pain modulation by Nmb in the peripheral nervous system has yet to be elucidated.

Three families of voltage-gated Ca^2+^ channels (VGCCs) are expressed in mammals, and each family has distinct biophysical properties. The Cav1 family L-type channels and the Cav2 family N-, P/Q-, and R-type channels are high voltage-activated (HVA) channels, whereas Cav3 family T-type channels are low voltage-activated channels 6, 7. Activation of T-type channels is characterized by small membrane depolarization, which is critically important for regulating neuronal excitability 8 and is therefore crucial to low-threshold exocytosis 9. At the molecular level, T-type channels include three main subtypes (Cav3.1/α_1_G, Cav3.2/α_1_H, and Cav3.3/α_1_I) 6 that are ubiquitously expressed in the brain and periphery but display distinctive expression patterns 10-12. In the context of primary afferent pain sensing, T-type channels have substantial impacts on action potential firing properties, including threshold, duration and frequency, and regulate neurotransmitter release 13, 14. Aberrant T-type channel function is clearly involved in pathological pain sensation 15, 16 by altering membrane excitability 7, 17, as shown by genetic 18, pharmacological 19, 20, and functional 21 analyses. Thus, T-type channels are considered potential therapeutic targets for pain treatment 7, 17.

In this study, we examined the role of Nmb in modulating T-type channels in TG neurons and dissected the detailed molecular components of the signalling pathway that elicit the nociceptive effects of Nmb. Our findings suggest that Nmb binding to NmbR triggers activation of the βγ subunit of G_q_ proteins and downstream AMPK/PKA. This signalling pathway stimulates Cav3.2 channels to increase *I*_T_ and subsequently enhances neuronal excitability, contributing to TG neuronal mechanical hypersensitivity.

## Materials and Methods

### Dissociation of TG neurons

All the procedures performed in this study were carried out in accordance with NIH guidelines and were approved by the Institutional Animal Care and Use Committee of Soochow University. TG neurons were dissociated as previously described 22, 23. Briefly, TGs were harvested from 8- to 10-week-old ICR mice of either sex, cut into small pieces and treated with 2 mg/mL collagenase D (Roche) for 20 min followed by 1.5 mg/mL trypsin (Sigma) at 37 °C for 15 min. Cells were dissociated via gentle trituration with fire-polished sterilized Pasteur pipettes and centrifuged through a 15% BSA gradient to remove debris and nonneuronal cells. TG neurons were resuspended in neurobasal A (Thermo Fisher) supplemented with B27 (Thermo Fisher) and transferred onto Matrigel-coated coverslips. Patch clamp recordings were conducted 2 to 6 h after plating. In shRNA-treated knockdown experiments, TG cells were replated onto coverslips 48 h after infection.

### Electrophysiology

Standard whole-cell recordings were made at room temperature (22-23 °C) as described previously 24, 25. Recording electrodes were pulled from borosilicate glass microcapillary tubes (Sutter Instruments) and had resistances from 3 to 5 MΩ when filled with internal solution. Recordings were obtained using a MultiClamp 700B amplifier (Axon Instruments). Data were digitized on a personal computer using a Digidata 1440 interface under the control of the pClamp10.2 acquisition package (Molecular Devices). Data were acquired at 20 kHz with a low-pass Bessel filter at 2-5 kHz. Series resistance was compensated to 80%. The internal solution used for T-type channel current recordings contained 110 mM CsCl, 4 mM Mg-ATP, 0.3 mM Na_2_-GTP, 25 mM HEPES, and 10 mM EGTA, adjusted to pH 7.4 with CsOH; 295 mOsm. The external solution used for T-type channel current recordings contained 5 mM BaCl_2_, 140 mM TEA-Cl, 10 mM HEPES, 0.5 mM MgCl_2_, 5 mM CsCl, and 5.5 mM glucose, adjusted to pH 7.35 with TEA-OH; 305 mOsm. In experiments for Kv current recordings, the extracellular solution contained 140 mM choline-Cl, 1 mM MgCl_2_, 0.03 mM CaCl_2_, 5 mM KCl, 10 mM HEPES, and 10 mM glucose, adjusted to pH 7.4 with KOH; 310 mOsm. The intracellular pipette solution contained 140 mM KCl, 1 mM MgCl_2_, 0.5 mM CaCl_2_, 3 mM Mg-ATP, 0.3 mM Na_2_-GTP, 10 mM HEPES, and 5 mM EGTA, adjusted to pH 7.4 with KOH; 295 mOsm. The two kinetically distinct Kv currents in small TG neurons were separated by the two-step voltage protocol as previously described 22, 26. The external solution for Nav current clamp recordings contained 2 mM KCl, 2 mM MgCl_2_, 25 mM HEPES, 2 mM CaCl_2_, 128 mM NaCl, and 30 mM glucose, adjusted to pH 7.4 with NaOH; 305 mOsm. The internal solution contained 10 mM NaCl, 110 mM KCl, 4 mM Mg-ATP, 0.3 mM Na_2_-GTP, 25 mM HEPES, and 2 mM EGTA, adjusted to pH 7.3 with KOH; 295 mOsm. Nmb was applied to a patched cell by an air-pressure microinjector (Pneumatic PicoPump, model PV830, WPI).

### Western blot analysis

Western blot analysis was performed as previously described 27, 28. Briefly, protein samples of mouse TGs were subjected to 10% SDS-PAGE and transferred to polyvinylidene difluoride membranes (Invitrogen, CA). The membranes were blocked with 5% skim milk in Tris-buffered saline containing 0.1% Tween-20 (TBST) and probed with antibodies against Nmb (rabbit, 1:1000, Abcam, Cat. No. ab191499), Cav3.1 (rabbit, 1:1000, Alomone, Cat. No. ACC-021), Cav3.2 (mouse, 1:1000, Novus, Cat. No. NBP1-22444), Cav3.3 (rabbit, 1:1000, Alomone, Cat. No. ACC-009), NmbR (rabbit, 1:500, Sigma, Cat. No. SAB4502914), phospho-AMPKα1 (rabbit, 1:500, Abcam, Cat. No. ab92701), AMPKα1 (rabbit, 1:600, Abcam, Cat. No. ab110036), Gαq/11 (mouse, 1:600, Santa Cruz Biotechnology, Cat. No. SC-365906), and Gβ (mouse, 1:500, Santa Cruz Biotechnology, Cat. No. SC-515822). The blots were then exposed to an anti-GAPDH antibody (rabbit, 1:5000, Cell Signaling Technology, Cat. No. #2118) as a loading control. After washing with TBST, the membranes were incubated with a peroxidase-conjugated secondary antibody. The protein bands were detected with enhanced chemiluminescence (GE Healthcare, Amersham). The membranes were imaged using a ChinX Bio-Imaging System (Shanghai, China), and densitometric analysis was conducted using the Quantity-One program (Bio-Rad).

### Transient expression in HEK293 cells

HEK293 cells (ATCC, VA) were cultured in DMEM/F-12 medium (Invitrogen) supplemented with 10% FBS, 100 U/mL penicillin G, and 0.1 mg/mL streptomycin using standard techniques 25. Human NmbR cDNA (Origene, MD) was subcloned into pCMV6-AC-GFP. The full-length α_1_ subunits of human Cav3.1 (α_1_G), Cav3.2 (α_1_H), and Cav3.3 (α_1_I) (kindly provided by Dr Terrance P. Snutch, University of British Columbia, Canada) were cloned into the pcDNA3 vector (Invitrogen). Cells were transfected using Lipofectamine 3000 reagent (Invitrogen) according to the manufacturer's protocol.

### Adenovirus transfection

RNA interference delivered by recombinant adenoviruses was conducted as previously described 27-29. Sequences of the short hairpin RNAs (shRNAs) for Gq and Gβ were designed based on GenBank accession numbers NM_008139.5 and NM_008142.4, respectively, and the optimal shRNA sequences (Gq: 5'-GCAAGAGCACCTTCATCAAGC -3'; G_β_: 5'-GCATCTGGCAAAGATTTATGC-3') were selected. Scrambled sequences (Gq: 5'-ACTCACTAGCACTCAGAGAGC-3'; G_β_: 5'-CTGATGTATCGATCACATGAG-3') were synthesized and employed as negative controls. The recombinant adenoviral shuttle plasmid pAdTrack-CMV-GFP carrying Gq shRNA (Ad-Gq-shRNA) or G_β_ shRNA (Ad-G_β_-shRNA) was constructed by Shanghai GeneChem Co., Ltd. (China). The resulting vector was linearized via PmeI digestion and cotransformed into *E. coli* BJ5183 cells along with the adenoviral backbone plasmid pAdEasy-1. After 48 h, small neurons with robust green fluorescence were selected for recordings. The shRNA-mediated knockdown efficiency was assayed by immunoblotting.

### Immunohistochemistry

Staining was performed as previously described 23, 28, 29. Briefly, mouse TG samples were serially sectioned into 15 μm thick slices using a CM 1950 cryostat (Leica Biosystems, Germany). The sections were blocked with 5% normal goat serum, 1% bovine serum albumin, and 0.2% Triton X-100 in PBS for 2 h and then probed with antibodies against NmbR (rabbit, 1:300, Sigma, Cat. No. SAB4502914), CGRP (mouse, diluted 1:1000, Abcam, Cat. No. ab81887) and NF-200 (mouse, diluted 1:600, Sigma, Cat. No. SAB4200811). After washing with PBS, the TG sections were subsequently visualized via incubation with Cy3-conjugated donkey anti-rabbit IgG (diluted 1:300, Merck Millipore, Cat. No. AP182C), FITC-conjugated donkey anti-mouse IgG (diluted 1:400, Sigma, Cat. No. AP192F), or FITC-coupled IB_4_ (diluted 1:500, Sigma, Cat. No. L2895). Fluorescence micrographs were taken on a Nikon Eclipse 104c fluorescence microscope (Japan) equipped with a charge-coupled device (CCD) camera (CoolSNAP, Photometrics).

### PKA activity assay

PKA enzymatic activity in cell lysates was measured using the Pep Tag assay for nonradioactive detection of PKA (Promega, Madison, WI, USA) as described in our previous studies. Briefly, TG cells were cultured in 12-well plates and preincubated with BIM23042 (0.5 μM) or compound C (10 μM) followed by exposure to 100 nM Nmb for 30 min. TG cells were washed with ice-cold phosphate-buffered saline and placed on ice. PKA was extracted from the cell lysates with cold PKA extraction buffer (25 mM Tris-HCl, pH 7.4, 0.5 mM EDTA, 0.5 mM EGTA, 10 mM β-mercaptoethanol, 1 μg/mL leupeptin and 1 μg/mL aprotinin). The assay was conducted according to the manufacturer's instructions.

### Behavioural tests

Mice were housed on a 12 h light/dark cycle, maintained in a humidity- and temperature-controlled room with water available ad libitum and were acclimated to the experimenter for 2 d before the first behavioural observations. Experimenters were blinded to the treatment or genotypes of the mice. As described previously 30, 31, an Orofacial Stimulation Test (Ugo Basile, Italy) was used for the measurement of hypersensitivity to the mechanical stimulation of the trigeminal area. Briefly, the mice initially underwent one week of adaptation trainings after a 12 h fasting food period. Each mouse was then placed in a cage in which there was an Orofacial Stimulation Test system. After the mouse was given 10 min to familiarize itself with its environment, the drinking window was opened and the testing mouse was subsequently timed for 10 min to allow drinking the milk. The contact number and total duration of time the mouse spent acquiring the reward were analyzed with the ORO software.

For experiments that several time points are needed to be checked in 24 h, *von* Frey test was used, as orofacial stimulation test can be checked only once a day 31, 32. For the *von* Frey test, the mice were placed in metal mesh boxes and allowed 30 min for habituation before examination. Mechanical hypersensitivity was assessed as described in previous studies 31, 33. In brief, the threshold was assessed with a set of *von* Frey hairs (0.02-2.56 g, Stoelting) applied to the whisker pad in an ascending series of trials, with three separate *von* Frey stimulations applied per series. The threshold was determined when mice moved their heads away briskly and strongly from at least one of the three stimuli. Dixon's up-down method was used to determine the 50% probability of escape threshold 33. For thermal test, a heat stimulus was applied to the maxillary whisker pad skin by radiant heat (IITC Life Science). Heat sensitivity was expressed as head-withdrawal latency (HWL). The radiant heat intensity was adjusted so that basal HWL is between 10-15 s, with a cut-off of 18 s to prevent tissue damage. Chronic inflammatory pain was induced by subcutaneous injection of 20 μL of complete Freund's adjuvant (CFA) into the left side of the buccal pad. The drugs (Nmb, BIM23042, Z941, or TTA-P2) were applied through a percutaneous approach by injecting the TG with a 30 G needle inserted through the infraorbital foramen, infraorbital canal and foramen rotundum. The tip of the needle terminated at the medial part of the TG, and a volume of 5 μL reagent was slowly delivered 31, 34. Mice received intra-TG injection of 5'-cholesteryl-modified and 6-FAM-modified Cav3.2 siRNA (Cav3.2-siRNA, 5 μg) or a scrambled negative control siRNA (NC-siRNA) (RiboBio, Guangzhou) 2 d after the CFA injection. siRNA was mixed with polyethyleneimine (PEI, Fermentas Inc.) for 10 min before being delivered. PEI was used as a delivery vehicle to prevent degradation and enhance cell membrane penetration of siRNAs 35, 36.

### Drugs and administration

Unless otherwise indicated, all pharmacological agents were purchased from Sigma. The QEHA (QEHAQEPERQYMHIGTMVEFAYALVGK) and SKEE peptides (SKEEKSDKER WQHLADLADFALAMKDT) 24, 37, 38 were synthesized by GenScript Corporation. Stock solutions of Nmb, NiCl_2_, GDP-β-S, cholera toxin (CTX), pertussis toxin (PTX), and PKC 19-36 were prepared in distilled deionized water. Stock solutions of phorbol 12-myristate 13-acetate (PMA), BIM23042 (Tocris Bioscience), rofecoxib, U73122, compound C, TTA-P2 (Alomone Labs), Z941 (kindly provided by Dr Terrance P. Snutch, University of British Columbia, Canada), KT-5720, and nifedipine were prepared in dimethyl sulfoxide (DMSO).

### Statistical analyses

Values are expressed as the mean values ± SEMs. Statistical significance was assessed using GraphPad Prism 6.0 (Synergy Software) or SPSS 16.0 (SPSS) software by one-way analyses of variance (ANOVA) with the post hoc Bonferroni test or by two-tailed t test for comparing two groups. Two-way ANOVA with repeated measures was used to analyse the differences in the ET to mechanical stimuli. p < 0.05 was considered statistically significant. The statistical analyses for individual experiments are described in the figure legends. The Nmb concentration-response curve data were fitted to the Hill equation: I/I_control_=1/(1+10^(log EC50-X)^*n*), where EC50 represents the concentration of agonist eliciting a 50% maximal response, X is the decadic logarithm of the agonist concentration, and *n* is the Hill coefficient. The plots of voltage-dependent activation and steady-state inactivation were fitted by the Boltzmann equation.

## Results

### Nmb enhances *I*_T_ in TG neurons

**Figure 1:**
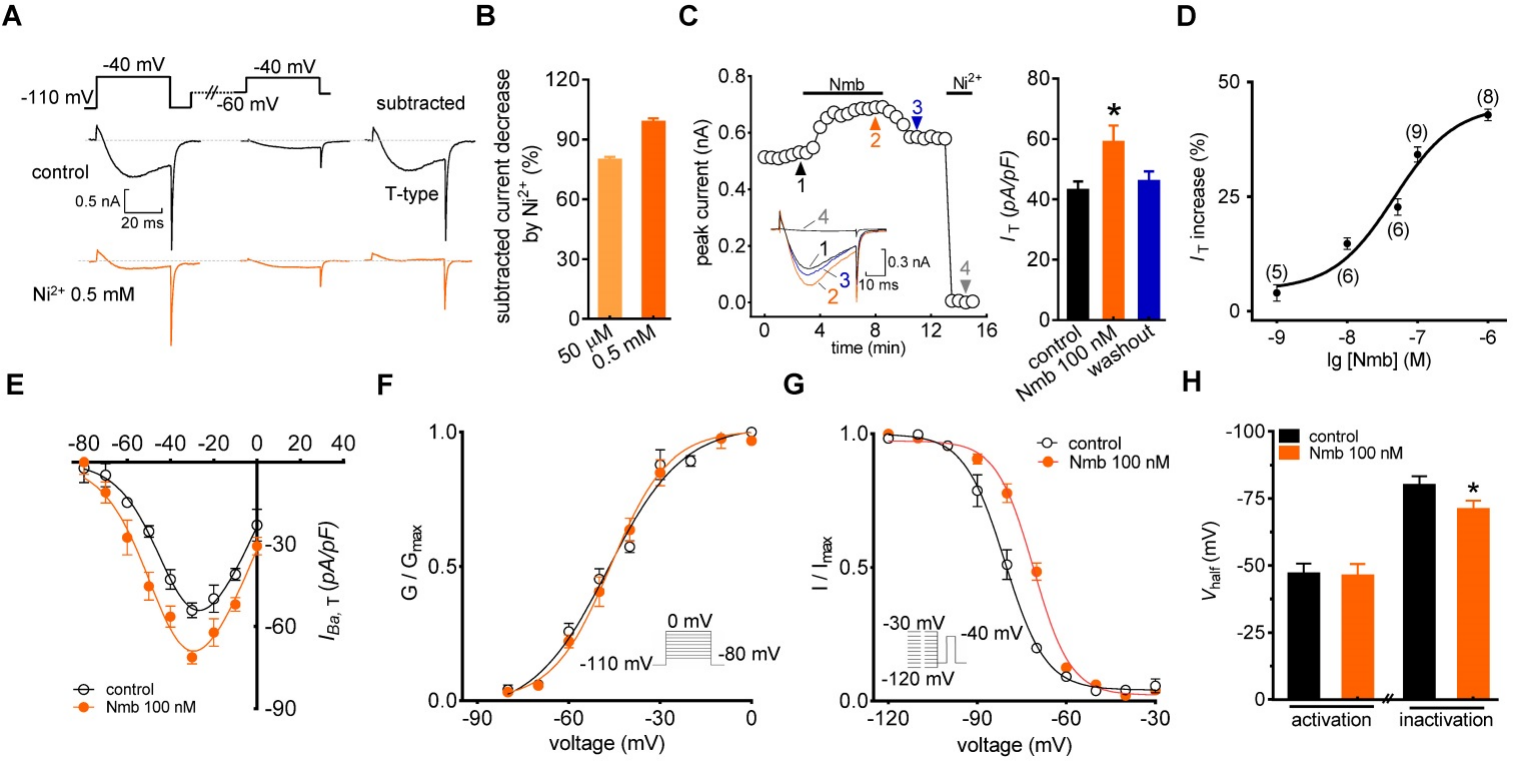
** Nmb enhances* I*_T_ in TG neurons. A,*** left panel*, representative recording from small TG neurons before and after 0.5 mM NiCl_2_ (Ni^2+^) application. Currents were recorded by a 40 ms depolarizing step pulse from the holding potential of -110 mV or -60 mV to -40 mV. *Inset*: remaining current (*I*_T_) after off-line subtraction. **B,** summary data indicate the inhibition of Ni^2+^ at 50 µM (*n* = 5) or 0.5 mM on *I*_T_ (*n* = 5). Ni^2+^ at 0.5 mM completely blocked *I*_T_ in TG neurons. **C,** time course (*left panel*) and summary data (*right panel*) show the effect of 100 nM Nmb on *I*_T_ (*n* = 9). *Inset*s indicate the exemplary current traces. The Arabic numbers represent points used for representative traces. Ni^2+^ (0.5 mM) was applied at the end of each voltage-clamp recording to determine the current baseline. *I*_T_ were then calculated by digitally subtracting currents after Ni^2+^-treatment from those before application of NiCl_2_. This protocol is used for calculation of pure *I*_T_ amplitude in all recordings. *p < 0.05 *versus* control, two-tailed *t* test. **D,** dose-dependent effects of Nmb on *I*_T_. Solid line represents the sigmoidal dose-response fits. Numbers in *parentheses* denote *n* cells tested at each concentration. **E,** current-voltage (*I-V*) plots show the effect of 100 nM Nmb on the current density of T-type channels at each voltage (*n* = 8). Currents were elicited by test pulses that range from -80 mV to 0 mV in increments of +10 mV. **F-G,** application of 100 nM Nmb had no significant effect on the voltage-dependent activation curve (*F*), but shifted the steady-state inactivation curve towards a depolarizing direction (*G*). *Inset*s, stimulation protocols. **H,** summary data show respective effects of Nmb (100 nM) on *V*_half_ of the activation (*n* = 9) and inactivation (*n* = 9) curves indicated in panels *F* and *G*, respectively. *p < 0.05 *versus* control, two-tailed *t* test.

In the current study, we sorted adult mouse TG neurons according to soma diameter into small- (≤ 25 μm) and medium-sized (25 to 35 μm) groups and performed whole-cell recording for only neurons in the small group, as they play critical roles in the nociceptive pathway 22, 39. We used the following protocol to dissect low-voltage activated (LVA) T-type channel currents, building on the knowledge that the majority of T-type channels are inactivated at more depolarizing holding potentials 40. First, we blocked most, if not all, high-voltage activated (HVA) Ca^2+^ channel currents by bath-applying a cocktail of channel blockers, including 5 μM nifedipine (L-type channel blocker), 0.2 μM ω-conotoxin MVIIC (N- and P/Q-type channel blocker), and 0.2 μM SNX482 (R-type channel blocker) 35, 41. Next, we recorded Ba^2+^ currents at -40 mV for 40 ms by depolarization from either -60 mV or -110 mV holding potential (*V*_h_). Current was then calculated by digitally subtracting currents measured from -60 mV *V*_h_ from those measured from -110 mV *V*_h_ (Figure 1A). This eliminated the residual HVA channel current that was not blocked by the cocktail of blockers 39, 42. The remaining inward current after subtraction was dramatically decreased by 50 μM T-type channel blocker NiCl_2_ (79.2%), and was almost completely abolished by 0.5 mM NiCl_2_ (Figure 1B). Moreover, to accurately obtain the pure *I*_T_ amplitude, 0.5 mM NiCl_2_ was applied at the end of each patch clamp recording to determine the current baseline. Currents through T-type channels (*I*_T_) were further calculated by digitally subtracting currents after NiCl_2_ treatment from those before NiCl_2_ application. As shown in Figure 1C, application of 100 nM Nmb to small TG neurons significantly increased the peak amplitude of *I*_T_ (34.2 ± 1.9%), and this effect was partially reversed when Nmb was removed by washing. Further addition of 0.5 mM NiCl_2_ at the end of recording almost abrogated the *I*_T_ (Figure 1C). Dose-response relationships were then examined, and by fitting the data with a sigmoidal *Hill* equation, we observed an EC_50_ of 45.3 nM (Figure 1D). Examination of the biophysical properties of *I*_T_ modified by 100 nM Nmb showed that Nmb downward shifted the current-voltage relationship curve (Figure 1E). Furthermore, we observed an ~7.3 mV depolarizing shift in steady-state inactivation (*V*_half_ from -80.1 ± 2.5 mV to -72.8 ± 1.3 mV) but no significant shifts in activation properties (*V*_half_ from -47.1 ± 1.9 mV to -46.3 ± 2.2 mV) (Figure 1F-H).

### NmbR mediates the Nmb-induced *I*_T_ increase

**Figure 2:**
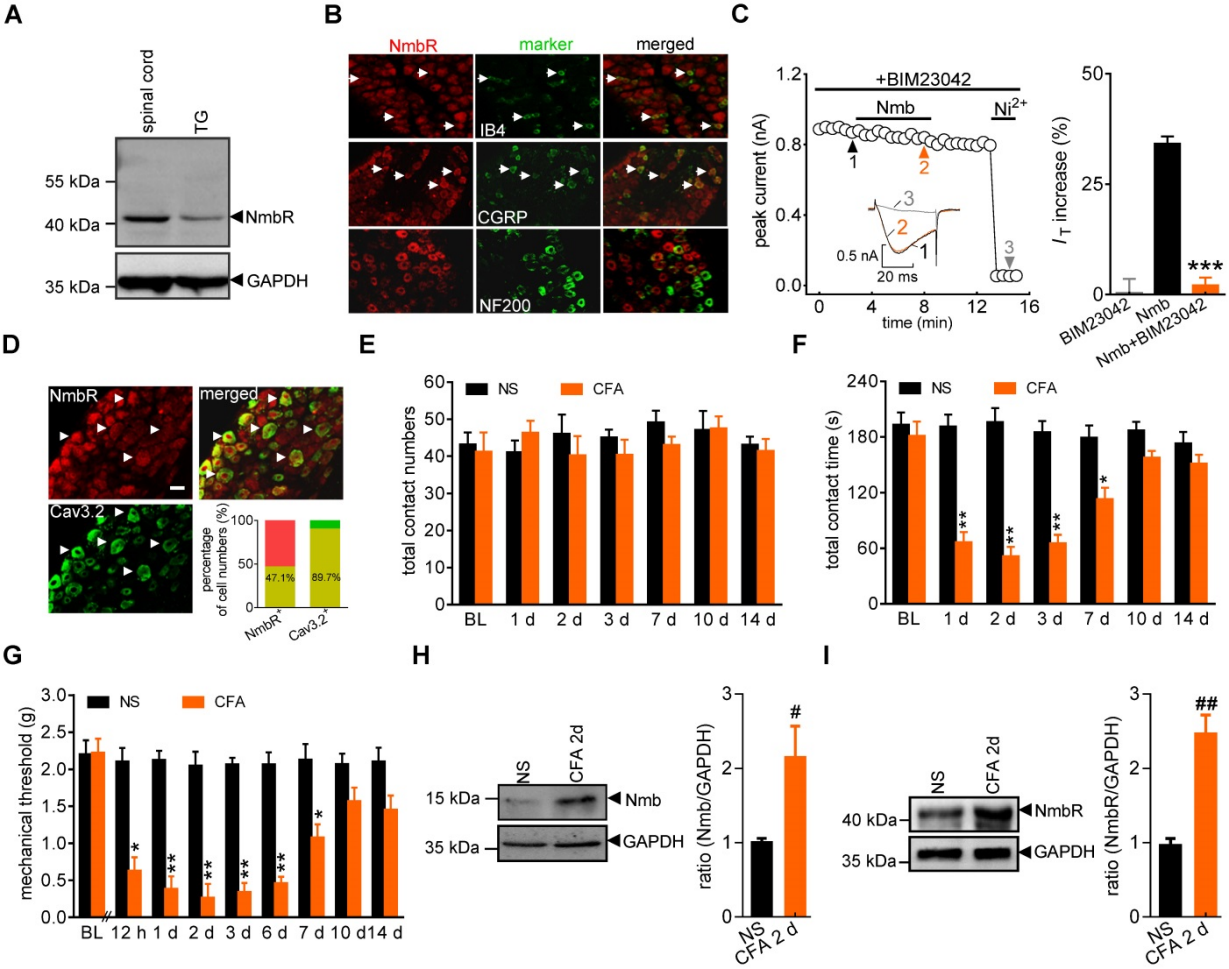
** NmbR mediates the Nmb-induced *I*_T_ increase. A,** western blot analysis of NmbR in mouse TGs. A prominent band was also observed in the spinal cord of mice. Blots depicted are representative of three independent experiments. **B,** double immunostaining of NmbR with calcitonin gene-related peptide (CGRP), isolectin B4 (IB_4_), or neurofilament 200 (NF200) in mouse TG sections. Arrows in white indicate the co-localization. Scale bar, 30 µm. **C,** time course (*left panel*) and summary data (*right panel*) show the effects of 100 nM Nmb on *I*_T_ in TG neurons pretreated with BIM23042 (0.5 µM) (*n* = 7). Bath application of 0.5 µM BIM23042 alone did not affect *I*_T_ (*n* = 6). *Inset*s indicate the exemplary current traces. The Arabic numbers represent points used for representative traces. ***p < 0.001 *versus* Nmb, two-tailed *t* test. **D,** double immunostaining of NmbR with Cav3.2 in TG neurons of naïve mice. Arrows in white show the colocalization. Scale bars, 30 µm. *Insets* indicate the percentage of double-labeled neurons in NmbR^+^ or Cav3.2^+^ neurons. **E,** total contact number of orofacial operant test with mechanical stimulation was comparable between normal saline (NS)- and CFA-treated mice. BL, baseline. **F,** total contact time was significantly decreased from 1 to 7 d after CFA injection, compared to NS group. *P < 0.05, **P < 0.01 versus NS, two-way ANOVA followed by Bonferroni post hoc test. **G,** mechanical hypersensitivity induced by CFA injection. Decreased escape threshold was observed at the 12th h after CFA injection and lasted to ~7 d (*n* = 9 mice per group). *p < 0.05 and **p < 0.01 *versus* normal saline (NS) at the corresponding time point, two-way ANOVA followed by Bonferroni post hoc test. **H,** representative immunoblots show that Nmb expression was increased in the local inflamed tissue of mice at 2 d after CFA. Blots depicted are representative of three independent experiments. ^#^*p* < 0.05 versus NS, two-tailed *t* test. **I,** abundance of NmbR protein in mouse TGs at 2 d after NS- or CFA- treatment. Blots depicted are representative of three independent experiments. ^##^*p* < 0.01 versus NS, two-tailed *t* test.

It has been shown that NmbR is an endogenous receptor specific for Nmb 1. We therefore investigated the expression profile of NmbR in mouse TGs. Immunoblot analysis of TG protein lysates revealed that NmbR was endogenously expressed in mice (Figure 2A, Figure S1). Further immunostaining of TG sections indicated that NmbR mainly colocalized with calcitonin gene-related peptide (CGRP) and isolectin B4 (IB4), while it showed comparatively little colocalization with neurofilament 200 (NF200) (Figure 2B). Subsequently, we investigated the involvement of NmbR in the Nmb-mediated stimulatory effects on T-type channels. Treatment with BIM23042 (0.5 µM), a potent and specific antagonist of NmbR, alone had no significant effect on *I_T_* in small TG neurons (increase of 0.5 ± 2.9%; Figure 2C), while pretreatment with 0.5 µM BIM23042 prevented the 100 nM Nmb-induced increase in *I_T_* (increase of 2.1 ± 1.7%; Figure 2C). Cav3.2 is the predominant isoform of the T-type Ca^2+^ channel family found in primary sensory neurons and is involved in nociceptive processing 43, 44. As a functional interaction between T-type channels and NmbR exists, we then determined whether Cav3.2 and NmbR are coexpressed in nociceptive TG neurons. Double staining showed that NmbR extensively colocalized with Cav3.2: 47.1% of NmbR^+^ neurons expressed Cav3.2; notably, 89.7% of Cav3.2^+^ neurons expressed NmbR (Figure 2D). We further analysed the NmbR protein abundance under conditions of chronic inflammatory pain. Orofacial stimulation tests showed that total contact number was not significantly changed after complete Freund's adjuvant (CFA) injection, and no significant difference was found between normal saline and CFA groups (Figure 2E). However, the total contact time was significantly reduced in mice 1 d after CFA treatment, while the saline-injected mice were not changed (Figure 2F). The time course of CFA-induced mechanical allodynia reflected by the reduction of contact time is comparable to the test by *von* Frey filaments (Figure 2G, lasted for ~7 d), suggesting both behaviour tests are reliable. Moreover, immunoblot analysis showed that Nmb protein was highly expressed in the local inflamed tissue 2 d after CFA injection (Figure 2H, Figure S2), and NmbR was specifically upregulated in the TGs (Figure 2I, Figure S3). In contrast, the upregulation of Nmb and NmbR was not observed in saline injected control group.

### The Nmb-induced T-type channel response requires G_q_ proteins

**Figure 3:**
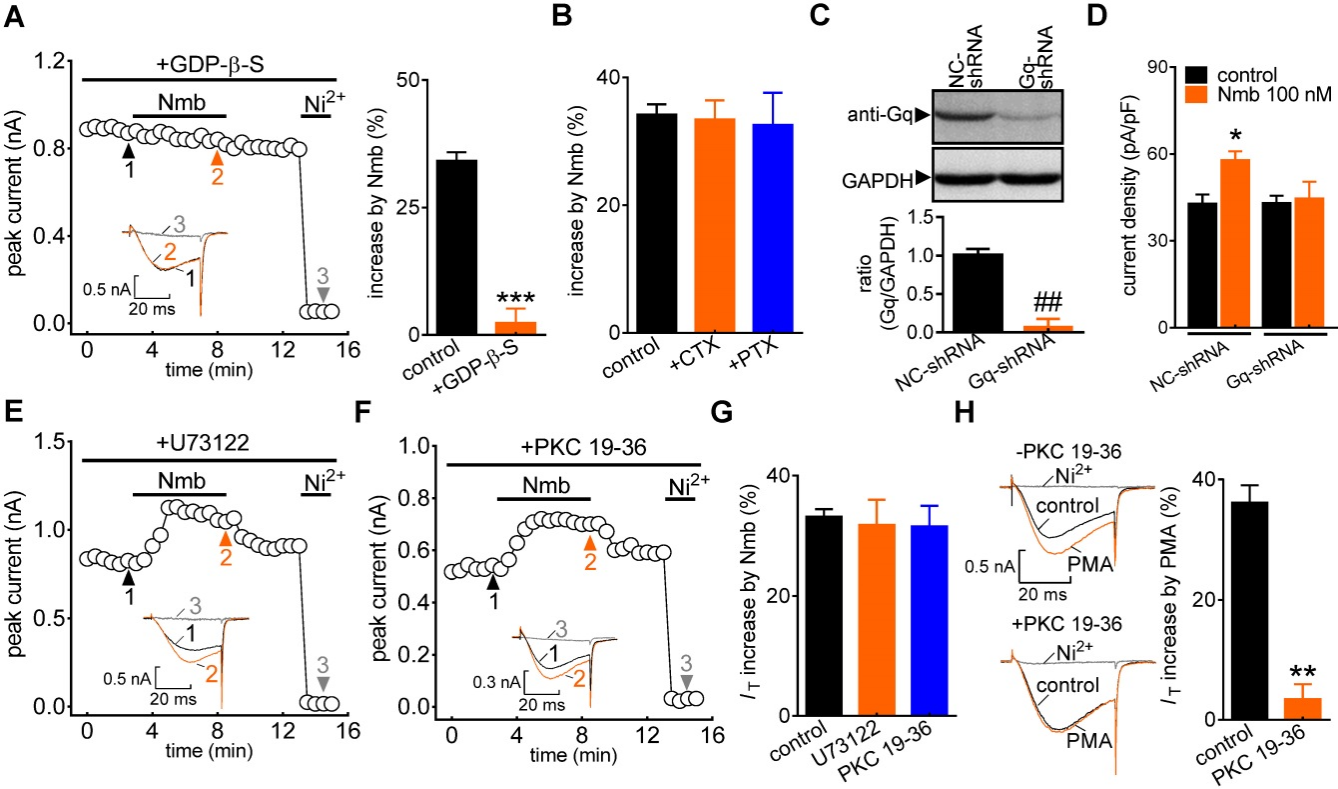
** The Nmb-induced *I*_T_ response requires G_q_ protein. A,** time course (*left panel*) and summary data (*right panel*) show the effect of 100 nM Nmb on *I*_T_ in the presence of GDP-β-S (1 mM, intracellular application) (*n* = 6). *Inset*s indicate the exemplary current traces. The Arabic numbers represent points used for representative traces. ***p < 0.001 *versus* control, two-tailed *t* test. **B,** summary data show the effect of 100 nM Nmb on *I*_T_ in TG neurons pre-incubated with pertussis toxin (PTX, 0.2 μg/mL, *n* = 8) and cholera toxin (CTX, 0.5 μg/mL, *n* = 8), respectively. **C,** protein expression level of G_q_ in TG cells transduced with Gq shRNA (Gq-shRNA) or negative control shRNA (NC-shRNA). GAPDH was used as the loading control. Blots depicted are representative of three independent experiments. ^##^*p* < 0.01 *versus* NC-shRNA, two-tailed *t* test. **D,** summary data show the effect of Nmb (100 nM) on *I*_T_ in TG neurons transduced with NC-shRNA (*n* = 10) or Gq-shRNA (*n* = 9). *p < 0.05* versus* NC-shRNA + control, one-way ANOVA followed by Bonferroni post hoc test. **E-F,** time course of* I*_T_ changes induced by 100 nM Nmb in TG neurons pre-incubated with U73122 (1 μM, *E*) or dialyzed with PKC 19-36 (10 μM,* F*). *Inset*s indicate the exemplary current traces. The Arabic numbers represent points used for representative traces. **G,** summary data indicate the effect of 100 nM Nmb on *I*_T_ indicated in panels *E* (*n* = 8) and *F* (*n* = 7), respectively.** H,** exemplary current traces and summary data indicate the effects of 5 μM PMA on *I*_T_ in the absence (*upper panel*, *n* = 7) or presence (*lower panel, n* = 5) of 10 μM PKC 19-36. **p < 0.01 *versus* control, two-tailed *t* test.

G protein-coupled receptors (GPCRs) mediate biological activities through either a classic heterotrimeric G protein-coupled or a G protein-independent (such as β-arrestin) mechanism 45. To investigate the potential involvement of G proteins, cells were dialysed with the nonhydrolysable GDP analogue GDP-β-S. Intracellular application of GDP-β-S (1 mM) completely abolished the Nmb-induced increase in *I*_T_ (increase of 2.3 ± 2.7%; Figure 3A), indicating that this increase requires G protein activation. We next studied the G protein subtypes involved in the NmbR-mediated response. Inactivating Gs via pretreatment with cholera toxin (0.5 μg/mL) did not affect the ability of Nmb to increase *I*_T_ (increase of 33.5 ± 2.9%; Figure 3B). Similar results were obtained with G_i/o_ inhibition by pretreating TG neurons with 0.2 μg/mL PTX (increase of 32.6 ± 5.1%; Figure 3B). The involvement of G_q/11_ in the NmbR response was then determined. Because there is still a lack of commercially available chemical G_q_ inhibitors, we employed adenovirus-mediated shRNA knockdown of Gq to study the modulation of *I*_T_ by Nmb in TG neurons. The protein expression level of G_q_ was significantly reduced in TG cells transduced with G_q_-shRNA (Figure 3C, Figure S4). The Nmb-induced *I*_T_ increase was completely prevented by knockdown of G_q_ (Figure 3D). These results indicate that the Nmb-induced increase in *I*_T_ requires G_q_ protein expression in TG neurons. PLC_β_ is regulated by G_q/11_ proteins and can activate the downstream effector PKC 46. We therefore pretreated TG neurons with the PLC inhibitor U73122 (3 μM) and found that the Nmb-mediated stimulatory effect was not affected by the PLC inhibitor (increase of 31.8 ± 4.2%; Figure 3E-G). As it has also been reported that PKC can be activated independently of PLC, we investigated whether the stimulatory effects of Nmb are mediated by PKC. Dialysis of TG neurons with 10 µM PKC 19-36, a pseudosubstrate peptide inhibitor of PKC, did not affect the *I*_T_ increase induced by Nmb (increase of 31.5 ± 3.5%; Figure 3F,G). The PKC 19-36 used in this study was demonstrated to be effective since it abrogated the increase in *I*_T_ induced by 5 μM PMA (Figure 3H).

### The G_βγ_-dependent AMPK/PKA pathway mediates the NmbR response

**Figure 4:**
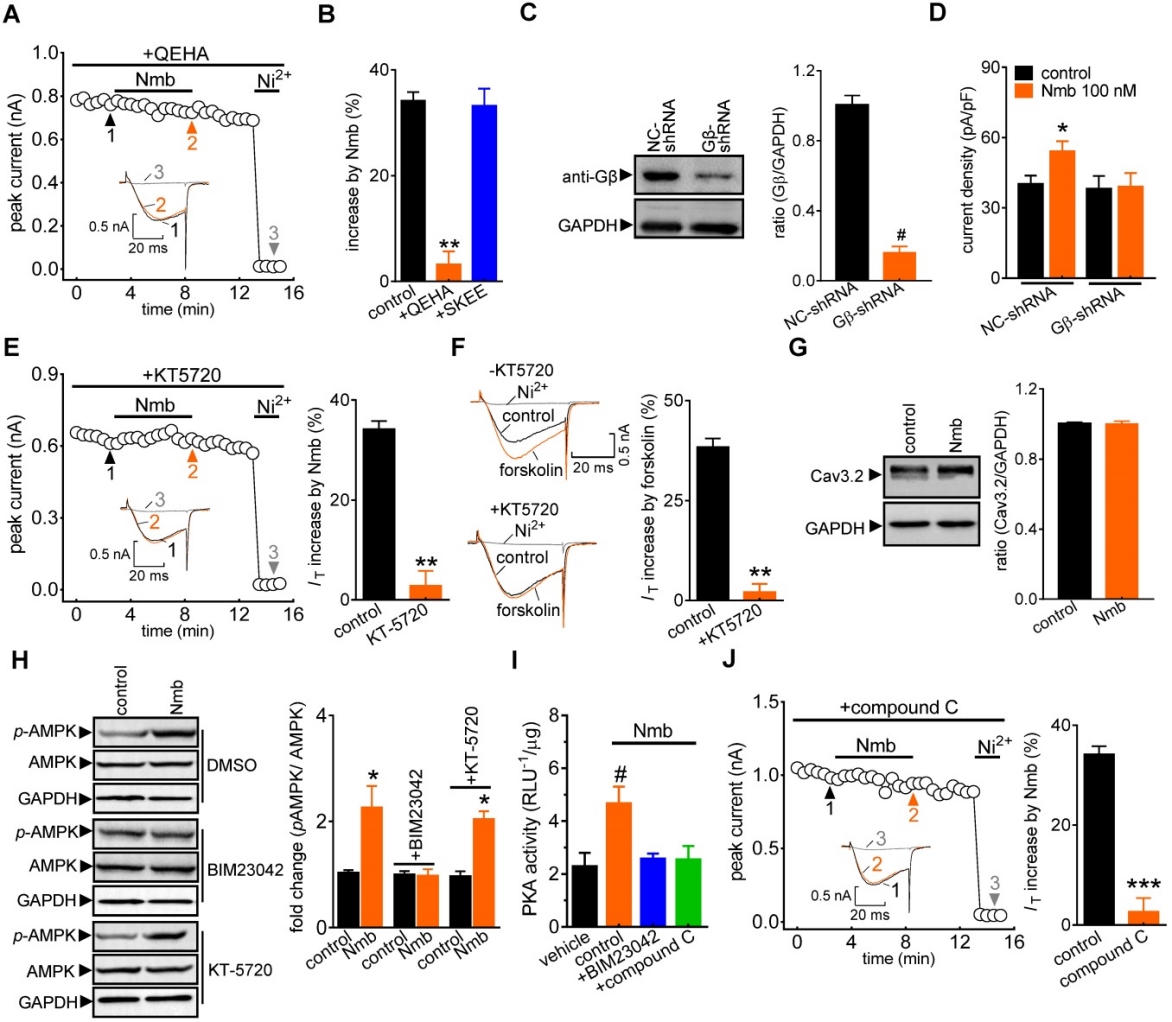
** The G_βγ_-dependent AMPK/PKA pathway mediates the NmbR response. A,** time course of* I*_T_ changes induced by 100 nM Nmb in TG neurons dialyzed with QEHA (10 μM).* Inset*s indicate the exemplary current traces. The Arabic numbers represent points used for representative traces. **B,** summary data indicate the effect of 100 nM Nmb on *I*_T_ in TG neurons dialyzed with QEHA (10 μM, *n* = 6) or SKEE (10 μM, *n* = 7). **p < 0.01 *versus* control, two-tailed *t* test.** C,** protein expression of G_β_ in cells transduced with G_β_ shRNA (G_β_-shRNA) or negative control shRNA (NC-shRNA). Blots depicted are representative of three independent experiments. ^#^*p* < 0.05 *versus* NC-shRNA, two-tailed *t* test. **D,** summary data show the effect of Nmb (100 nM) on *I*_T_ in TG neurons transduced with NC-shRNA (*n* = 9) or G_β_-shRNA (*n* = 8). *p < 0.05 *versus* NC-shRNA + control, one-way ANOVA followed by Bonferroni post hoc test. **E,** time course (*left panel*) and summary data (*right panel*) show the effect of Nmb (100 nM) on *I*_T_ in cells pre-incubated with KT-5720 (1 μM, *n* = 7). *Inset*s indicate the exemplary current traces. The Arabic numbers represent points used for representative traces. **p < 0.01 *versus* control, two-tailed *t* test. **F,** exemplary current traces and summary of results show the effect of forskolin (10 µM) on *I*_T_ in TG neurons in the absence (*n* = 7) or presence of KT-5720 (1 μM, *n* = 6). **p < 0.01 *versus* control, two-tailed *t* test.** G,** representative immunoblot and summary data show that treatment of TG cells with 100 nM Nmb did not affect the protein expression level of Cav3.2. GAPDH was used as the loading control. **H,** pretreatment of TG cells with 0.5 µM BIM23042, but not 1 μM KT-5720, attenuates the increased expression level of phosphorylated AMPK (*p*-AMPK) induced by 100 nM Nmb. GAPDH was used as the loading control. Blots depicted are representative of three independent experiments. *p < 0.05 *versus* control, two-tailed *t* test. **I,** summary data show the effect of 100 nM Nmb on PKA activity in TG cells pretreated with 0.5 µM BIM23042 or 10 µM compound C. All experiments were performed in triplicate with similar results.^ #^*p* < 0.05 *versus* vehicle, one-way ANOVA followed by Bonferroni post hoc test. **J,** time course (*left panel*) and summary of results (*right panel*) indicate the effect of 100 nM Nmb on *I*_T_ in TG neurons pretreated with compound C (10 µM, *n* = 7). *Inset*s show the exemplary current traces. The Arabic numbers represent points used for representative traces. ***p < 0.001 *versus* control, two-tailed *t* test.

Next, we determined the potential role of the βγ subunit (G_βγ_) of the G_q_ protein in the NmbR-mediated T-type channel response. Dialysis of small TG neurons with the G_βγ_ antagonist QEHA peptide (10 μM) prevented the Nmb-induced increase in *I*_T_ (increase of 3.2 ± 2.5%; Figure 4A,B), while intracellular infusion of SKEE (10 μM), the scrambled peptide of QEHA, elicited no such effects (increase of 33.2 ± 3.5%; Figure 4B). As a complementary test of our hypothesis, we also determined the effect of Nmb on T-type channels in G_β_-silenced TG neurons. Immunoblot analysis showed that G_β_ expression was significantly reduced in cells transduced with G_β_ shRNA (Figure 4C, Figure S5). Knockdown of G_β_ prevented the Nmb-induced T-type channel response (Figure 4D). Together, these results indicate that G_βγ_ of the G_q_ protein is required in the transduction pathway, leading to an increase in *I*_T_ induced by NmbR stimulation. Next, we investigated in detail the mechanism underlying the NmbR-mediated T-type channel response. We determined the role of PKA in the Nmb-induced response since stimulation of PKA modulates T-type channels 47. Pretreating TG neurons with KT-5720 (1 µM), a PKA inhibitor, completely abolished the 100 nM Nmb-mediated *I*_T_ response (increase of 2.8 ± 2.9%; Figure 4E). Furthermore, application of 10 μM forskolin, a PKA agonist, significantly increased *I*_T_ by 38.3 ± 2.1% in mouse TG neurons (Figure 4F); and this effect was abrogated by KT-5720 (1 µM) pretreatment. The treatment of 100 nM Nmb did not affect the Cav3.2 protein expression in TG cells as shown by immunoblot analysis (Figure 4G, Figure S6), excluding the possibility that PKA-induced* I*_T_ increase was through regulation of channel expression. As AMP-activated protein kinase (AMPK) can activate PKA 48, we next examined whether AMPK is necessary for Nmb-induced PKA activation. Western blot analysis showed that Nmb exposure in TG cells markedly increased the level of phosphorylated AMPK (*p*-AMPK; Figure 4H, Figure S7). Pretreatment of TG neurons with the NmbR inhibitor BIM23042 (0.5 µM) but not the PKA antagonist KT-5720 (1 µM) eliminated Nmb-induced AMPK activation (Figure 4H, Figure S7). In contrast, Nmb-induced increase in PKA activity was abrogated by pretreatment with the AMPK inhibitor compound C (10 µM), suggesting that AMPK acts as an upstream effector of PKA in the NmbR signalling pathway (Figure 4I). We then determined whether AMPK activation is actually involved in the Nmb-induced T-type channel response. Preincubation of TG neurons with 10 µM compound C precluded the Nmb-induced increase in *I*_T_ (increase of 2.6 ± 2.8%; Figure 4J). Taken together, these findings indicate that AMPK-dependent PKA is required for Nmb to exert its stimulatory effect on T-type channels in small TG neurons.

### Activation of NmbR selectively increases cloned Cav3.2 channel currents

**Figure 5:**
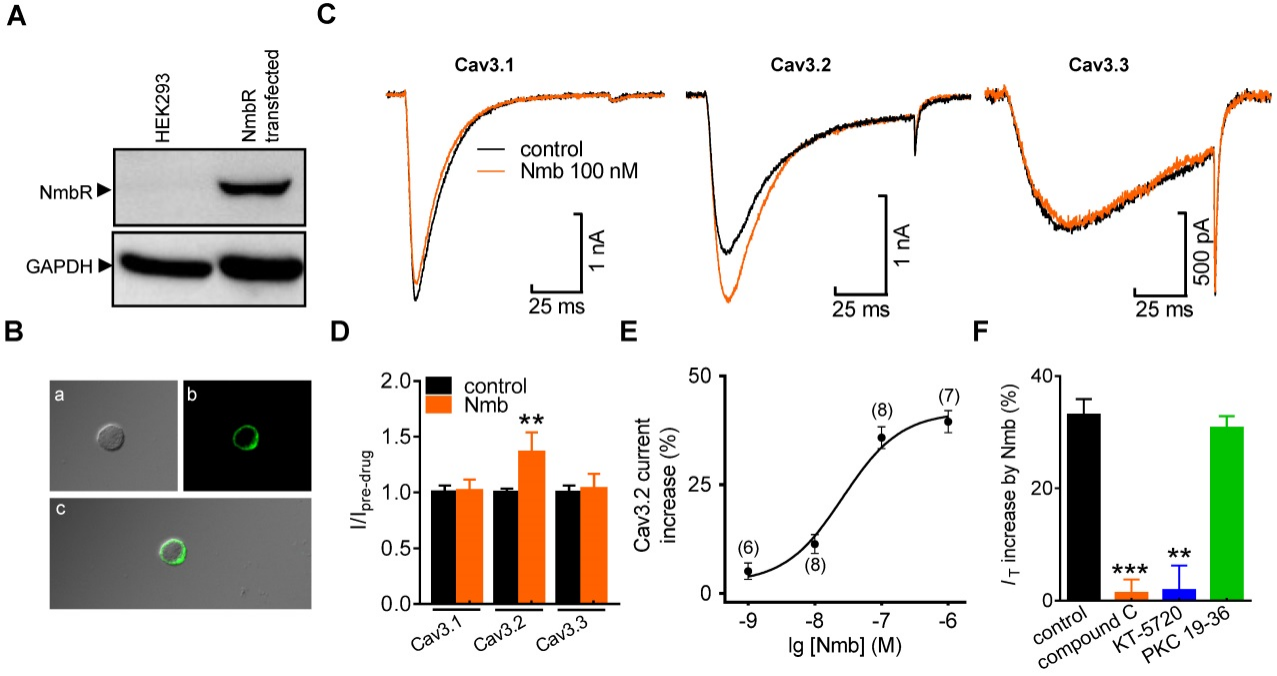
** Activation of NmbR stimulates recombinant Cav3.2 channels heterologously expressed in HEK293 cells. A,** western blot analysis of NmbR in HEK293 cells transiently transfected with *NmbR* cDNA. Blots depicted are representative of three independent experiments. **B,** membrane localization of NmbR in transfected HEK293 cells. Alphabets *a* through *c* in the diagram indicate the differential interference contrast (DIC, *a*), the EGFP fluorescent signals of NmbR (*b*), and the merged image (*c*), respectively. **C,** exemplary current traces show the effect of Nmb (100 nM) on Cav3.1 (α1G), Cav3.2 (α1H) and Cav3.3 (α1I) channel currents. Currents were elicited by a 100 ms depolarizing step pulse from the holding potential of -110 mV to -30 mV. **D,** summary data show the effect of 100 nM Nmb on Cav3.1 (*n* = 10), Cav3.2 (*n* = 9) and Cav3.3 (*n* = 7) channel currents. **p < 0.01 *versus* control, two-tailed *t* test. **E,** dose-dependent effects of Nmb on Cav3.2 channel currents. Solid line represents the sigmoidal dose-response fits. Numbers in *parentheses* denote *n* cells tested at each concentration. **F,** summary data show the effect of Nmb (100 nM) on Cav3.2 channel currents in cells pre-incubated with 10 µM compound C (*n* = 8), in cells pretreated with 1 µM KT-5720 (*n* = 9), and in cells dialyzed with 10 μM PKC 19-36 (*n* = 9), respectively. **p < 0.01 and ***p < 0.001 *versus* control, two-tailed *t* test.

Next, we determined the exact subunit of Cav3 involved in the NmbR-mediated *I*_T_ response. NmbR was not endogenously expressed in human embryonic kidney 293 (HEK293) cells (Figure 5A, Figure S8); we therefore coexpressed both the human cloned NmbR and Cav3 isoforms (Cav3.1, Cav3.2, or Cav3.3) into HEK293 cells. Western blot analysis using an antibody against NmbR showed a prominent band (Figure 5A, Figure S8), and immunostaining analysis suggested that NmbR was predominantly localized in the membrane (Figure 5B). Application of 100 nM Nmb robustly increased Cav3.2 channel currents (increase of 36.2 ± 3.1%), while neither the Cav3.1 (increase of 0.7 ± 1.2%) nor Cav3.3 channels (increase of 2.9 ± 1.6%) (Figure 5C,D) were affected. Further examination demonstrated that Nmb increased the Cav3.2 channel currents in a concentration-dependent manner (Figure 5E, EC_50_ of 47.6 nM). In addition, pretreatment with either 10 µM compound C (increase of 1.4 ± 2.9%) or 1 µM KT-5720 (increase of 1.9 ± 4.2%) completely abolished the NmbR-mediated change in Cav3.2 channel currents (Figure 5F), whereas dialysis of cells with PKC 19-36 elicited no such effect (increase of 30.8 ± 2.3%).

### Nmb increases TG neuronal excitability

**Figure 6:**
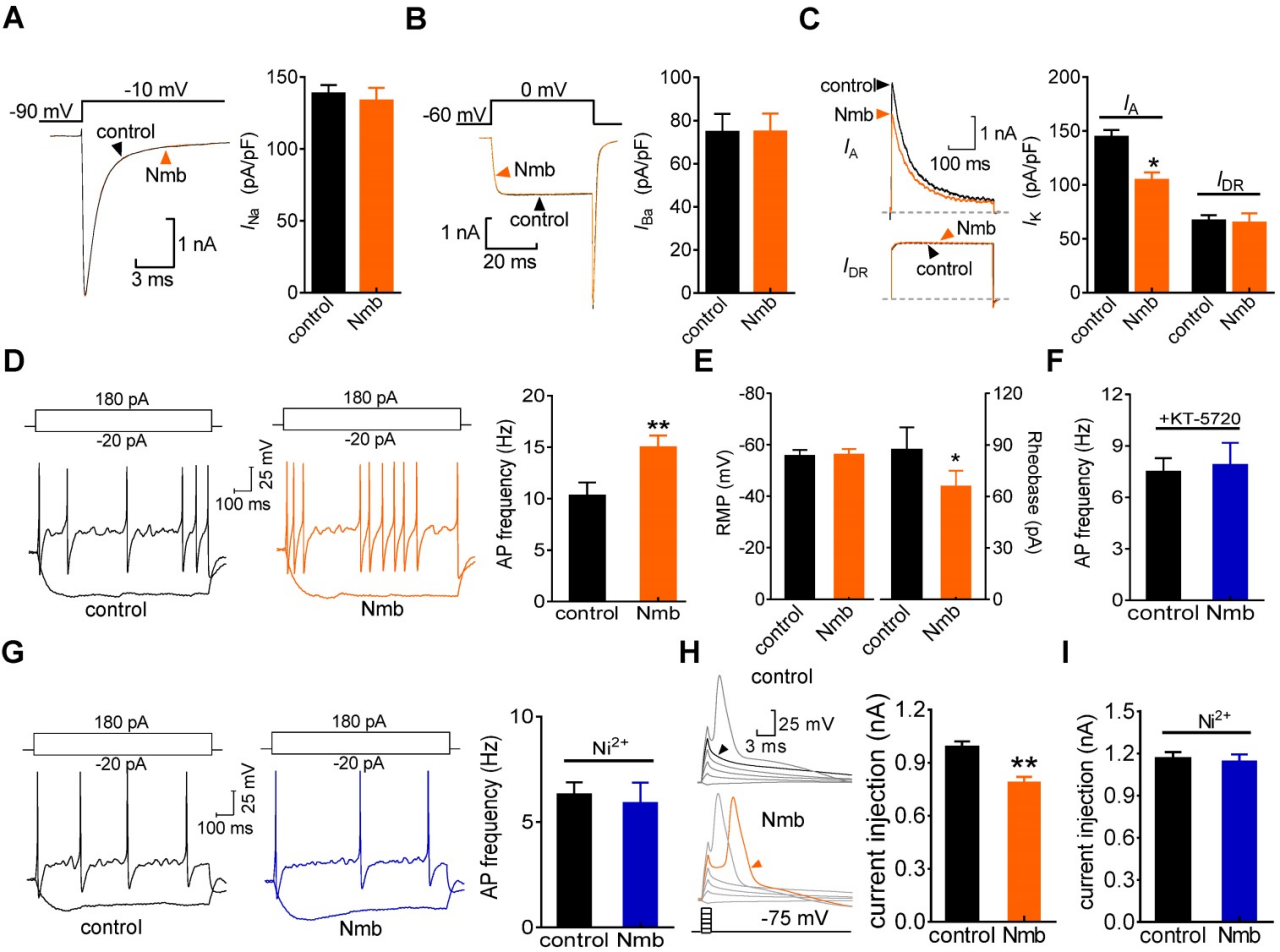
** Nmb increased neuronal excitability of TG neurons. A-C,** exemplary current traces (*left panel*) and summary data (*right panel*) show the effects of Nmb (100 nM) on voltage-gated Na^+^ channel (Na_v_) currents (**A**), high-voltage activated (HVA) Ca^2+^ channel currents (**B**), transient outward A-type K^+^ currents (*I*_A_, upper, **C**) and the sustained delayed-rectifier K^+^ currents (*I*_DR_, lower, **C**) in TG neurons, respectively. *p < 0.05 *versus* control, two-tailed *t* test. **D,** representative traces (*left panel*) and summary data (*right panel*) indicate that 100 nM Nmb markedly increased the action potential (AP) firing rate (*n* = 17). **p < 0.01 *versus* control, two-tailed *t* test. E, summary data indicate that Nmb at 100 nM had no effect on the resting membrane potential (RMP), but significantly decreased the rheobase of neuronal excitability (*n* = 17). *p < 0.05 *versus* control, two-tailed *t* test. **F,** summary data show that pretreating TG neurons with KT-5720 (1 μM) abolished the Nmb-induced response in AP firing rate (*n* = 12). **G,** representative traces (*left panel*) and summary data (*right panel*) show that NiCl_2_ (Ni^2+^) at 50 μM completely prevented the Nmb-mediated neuronal hyperexcitability (*n* = 11). **H,** *left panel*, representative traces from a Nmb-sensitive neuron. *right panel,* summary data indicate that the threshold was +0.98 nA in control and +0.79 nA in neurons treated with 100 nM Nmb. **p < 0.01 *versus* control, two-tailed *t* test. **I,** summary data show Ni^2+^ at 50 μM completely prevented the Nmb-induced lower threshold (*n* = 9).

We next determined the role of the Nmb-induced T-type channel response in regulating TG neuronal excitability. Nmb at 100 nM did not affect the whole-cell currents through voltage-gated Na^+^ channels (Nav) (Figure 6A). Initial analysis showed that the application of Nmb (100 nM) significantly decreased L-type channel currents (decrease of 19.6 ± 2.2%; Figure 6B). When 5 μM nifedipine was added to the external solution to block L-type channels, Nmb did not affect the remaining HVA Ca^2+^ channel currents (decrease of 1.3 ± 0.6%; Figure 6B). Furthermore, application of Nmb (100 nM) to TG neurons decreased the peak amplitude of the transient outward K^+^ channel currents (A-type currents or *I*_A_) (decrease of 22.3 ± 3.9%; Figure 6C), while the sustained delayed rectifier K^+^ channel currents (*I*_DR_) remained unaffected (decrease of 1.9 ± 1.6%; Figure 6C). Furthermore, in a bath solution containing 5 mM 4-aminopyridine (4-AP) and 5 μM nifedipine, Nmb at 100 nM significantly increased the action potential firing rate by 43.7 ± 3.7% in response to a 1-s current injection (Figure 6D). Additionally, the rheobase of Nmb-treated TG neurons was significantly lower than that of nontreated TG neurons (Figure 6E). Other membrane properties, including the AP threshold and resting membrane potential (Figure 6E), were not significantly changed. Pretreating TG neurons with 1 µM KT-5720 blocked the 100 nM Nmb-mediated increase in the AP firing rate, indicating that PKA is involved in this process (Figure 6F). To verify that Nmb-induced hyperexcitability occurred via increased *I*_T_, 50 μM NiCl_2_ was applied to the external solution and found to completely abolish the TG neuronal hyperexcitability induced by 100 nM Nmb (Figure 6G). These results suggest that Nmb acts to stimulate T-type channels and subsequently induce hyperexcitability in small TG neurons. To further test this hypothesis, a shorter (1 ms) current injection was applied to avoid contaminating the action potential waveform with the stimulus. Neurons were manually hyperpolarized to membrane potentials of -75 mV to maximize the number of T-type channels available for activation. With increasingly stronger current injections (+200 pA), the threshold current necessary to evoke an overshooting AP was determined for each neuron in the presence or absence of Nmb. In 15 of 19 cells tested, Nmb at 100 nM significantly lowered the threshold of neuronal excitability by ~21.7% (Figure 6H). Preincubation of TG neurons with NiCl_2_ (50 µM) completely prevented the decrease in the AP threshold induced by Nmb (Figure 6I).

### Involvement of T-type channels in Nmb-mediated pain hypersensitivity

**Figure 7:**
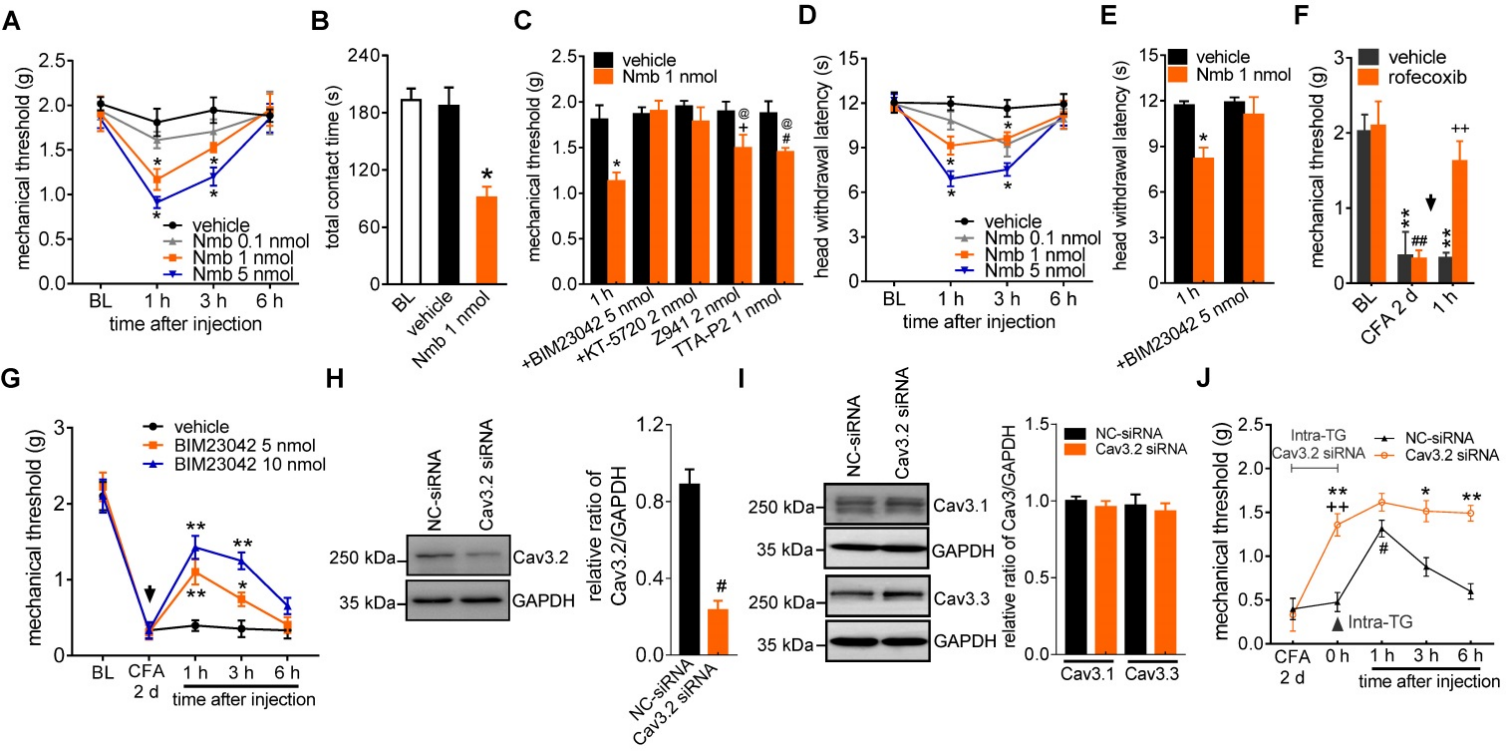
** Involvement of peripheral NmbR in mechanical pain hypersensitivity. A,** intra-TG injection of 1 nmol or 5 nmol of Nmb significantly induces mechanical hypersensitivity (*n* = 6 - 8 mice per group). *p < 0.05 *versus* vehicle, two-way ANOVA followed by Bonferroni post hoc test. BL, baseline. **B,** the total contact time assessed by orofacial operant test was significantly decreased 1 h after intra-TG injection of 1 nmol Nmb, compared to vehicle group. *p < 0.05 *versus* vehicle, one-way ANOVA followed by Bonferroni post hoc test. **C,** pretreatment of BIM23042 (5 nmol), KT-5720 (2 nmol), Z941 (2 nmol) or TTA-P2 (1 nmol) significantly attenuates 1 nmol Nmb-induced mechanical hypersensitivity (*n* = 8 - 10 mice per group). *p < 0.05 *versus* vehicle at 1 h, ^+^*p* < 0.05 *versus* Z941 + vehicle, ^#^*p* < 0.05 *versus* TTA-P2 + vehicle, one-way ANOVA followed by Bonferroni post hoc test. **D,** intra-TG injection of 1 nmol or 5 nmol of Nmb significantly induces heat hypersensitivity. *p < 0.05 *versus* vehicle, two-way ANOVA followed by Bonferroni post hoc test. **E,** intra-TG administration of BIM23042 (5 nmol) prevented 1 nmol Nmb-induced heat hypersensitivity (n = 6 - 8 mice per group). *p < 0.05 *versus* vehicle at 1 h, one-way ANOVA followed by Bonferroni post hoc test. **F,** rofecoxib (1 mg/kg administrated subcutaneously) reduced the mechanical allodynia produced by CFA. Behavioral analysis was performed at 2 d after CFA injection, and the effect of rofecoxib was assessed at 1 h after administration (n = 7 - 9 mice per group). **p < 0.01 *versus* basline (BL) in vehicle group, ^##^*p* < 0.01 *versus* basline (BL) in rofecoxib group, ^++^*p* < 0.01 *versus* vehicle at 1 h. **G,** intra-TG injection of BIM23042 (5 nmol) 2 d after CFA injection significantly suppresses the mechanical hypersensitivity in CFA-treated group (*n* = 7 - 9 mice per group). *p < 0.05 and **p < 0.01 *versus* vehicle, two-way ANOVA followed by Bonferroni post hoc test. **H-I,** intra-TG administration of Cav3.2 siRNA resulted in a significant decrease of Cav3.2 protein abundance in mouse TGs (*H*), while the expression level of Cav3.1 or Cav3.3 (*I*) remained unchanged. The blots shown are representative of three independent experiments. ^#^*p* < 0.05* versus* NC-siRNA, two-tailed *t* test. **J,** siRNA knock-down of Cav3.2 attenuates the BIM23042-induced alleviation of mechanical allodynia in CFA-treated mice (*n* = 8 - 10 mice per group). *p < 0.01 and **p < 0.01 *versus* NC-siRNA in CFA-treated mice;^ ++^*p* < 0.01 *versus* CFA 2 d; ^#^*p* < 0.05 *versus* 0 h point in NC-siRNA-treated mice, two-way ANOVA followed by Bonferroni post hoc test. The black arrow indicates the intra-TG injection of 5 nmol BIM23042.

To show the physiological functions of Nmb in pain hypersensitivity, we next investigated the contribution of NmbR signalling to behavioural signs of pain at the whole animal level. The mechanical threshold in response to *von* Frey filaments was first determined. Intra-TG application of 1 nmol or 5 nmol Nmb significantly induced profound hypersensitivity to acute mechanical pain, while Nmb at 0.1 nmol elicited no such effects. Nmb at 5 nmol showed stronger effect than that of 1 nmol and the effect started from 1 h, maintained at 3 h and recovered at 6 h (Figure 7A). Furthermore, orofacial operant behavioral assessment showed that the total contact time was significantly reduced in mice 1 h after intra-TG application of 1 nmol Nmb, while the vehicle-injected mice were not changed (Figure 7B). This effect was abolished by prior intra-TG injection of 5 nmol of the NmbR antagonist BIM23042 (Figure 7C) or 2 nmol of the PKA inhibitor KT-5720 (Figure 7C). Furthermore, intra-TG treatment with the potent T-type channel blocker TTA-P2 at 1 nmol prior to Nmb treatment significantly attenuated mechanical hypersensitivity induced by Nmb (Figure 7C). Similar results were observed with another potent small organic T-type channel blocker, Z941, at 2 nmol (Figure 7C). These results proved that NmbR-mediated hypersensitivity to acute pain is dependent on T-type channels *in vivo*. In addition, as shown in Figure 7D, the vehicle did not affect the heat hypersensitivity, whereas intra-TG injection of 1 nmol or 5 nmol of Nmb significantly decreased the head withdrawal latency (HWL) at 1 h; the effect maintained at 3 h. Intra-TG injection of 5 nmol of BIM23042 also blocked the heat hypersensitivity (Figure 7E). Next, we determined the role of NmbR signalling in a mouse model of CFA-induced inflammatory pain. After 2 d CFA treatment the mechanical threshold was significantly reduced from baseline. This CFA-induced mechanical allodynia was reversed by a selective COX-2 inhibitor rofecoxib (10 mg/kg) (Figure 7F), which validated the chronic inflammatory pain model 49. Moreover, pharmacological blockade of NmbR by intra-TG injection of 5 nmol or 10 nmol of the NmbR antagonist BIM23042 2 d after CFA treatment attenuated CFA-induced mechanical allodynia, and the effect was sustained for 3 h (Figure 7G). To further validate the Cav3.2 channel as an important cellular target for pain-alleviating effects of Nmb signaling in inflammatory pain, we administered intra-TG injection of siRNA specific for the Cav3.2 (Figure S9). Administration of Cav3.2-siRNA resulted in a significant down-regulation of Cav3.2 protein expression (Figure 7H and Figure S10), while the expression level of Cav3.1 or Cav3.3 remained unchanged (Figure 7I, Figure S11). Compared to the scramble siRNA control, local knockdown of Cav3.2 T-type channels led to a significantly decreased hypersensitivity of chronic inflammatory pain in mice (Figure 7J). Intriguingly, compared to Cav3.2 siRNA treatment alone, double treatment with 5 nmol BIM23042 and Cav3.2 siRNA showed no additive effect on mechanical sensitivity (Figure 7J), suggesting that NmbR and Cav3.2 T-type channels operate in the same signalling pathway. Collectively, these data demonstrate that Cav3.2 T-type channels are involved in NmbR-mediated pain hypersensitivity in CFA-induced inflammatory pain *in vivo*.

## Discussion

**Figure 8:**
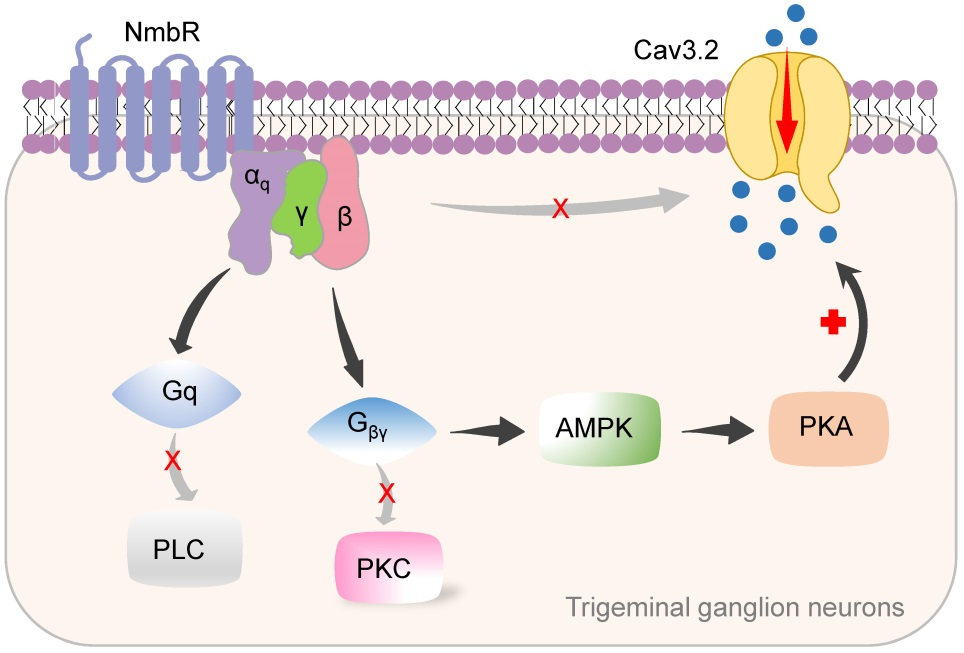
** Proposed mechanisms of NmbR signalling on Cav3.2 channels in TG neurons.** The binding of Nmb triggers NmbR to activate G_q_ protein and releases the G_βγ_ subunits. The released G_βγ_ dimer then stimulates AMPK activity, induces the activation of PKA and subsequently phosphorylates Cav3.2 channels to increase *I*_T_. Neither PLC/PKC nor direct binding of G_βγ_ to Cav3.2 channels is required for T-type channel response mediated by NmbR.

In this study, we identified a new signalling pathway underlying Nmb-induced hyperexcitability in small TG neurons. The binding of Nmb to NmbR triggers the release of the G_βγ_ subunits of Gq proteins and activates downstream AMPK and PKA. This signalling pathway plays a pivotal role in regulating Cav3.2 T-type channels and is involved in pain hypersensitivity *in vivo* (a schematic illustration is shown in Figure 8).

Distinct from G_q_-mediated stimulation of PLC and downstream PKC signaling, G_q_ has been shown to directly stimulate cation channels 46. Interestingly, in mouse small TG neurons, the G_q_ protein enhances NmbR-induced T-type channel activity through the actions of a common pool of G_βγ_ dimers 24, 27, 50. Our findings are based upon the following results: 1) dialysis of small TG neurons with GDP-β-S but not preincubation of neurons with PTX or CTX completely abolished the Nmb-mediated T-type channel response; 2) shRNA-mediated knockdown of Gq blocked the effect of Nmb; and 3) antagonism of G_βγ_ via intracellular application of the QEHA peptide blocked the Nmb-mediated response. It was previously shown that G_βγ_ (mainly the β2γ2 subtype) can directly interact with Cav3.2 to inhibit T-type channels 51, 52. However, in TG neurons, NmbR induced an increase in T-type channels, suggesting that a direct interaction between G_βγ_ and Cav3.2 is unlikely in this scenario. The insensitivity of T-type channels expressed in mouse TG neurons to G_βγ_ of the G_q_ protein could be due to different G_βγ_ subtypes or alternative splicing isoforms of Cav3.2 53. Indeed, the significant diversity of T-type channels is the result of Cav3 alternative splicing 12, 54, 55. Additionally, the possibility that an intermediate protein was involved in the observed G_βγ_-mediated response may not be excluded.

It has been demonstrated that G_βγ_ subunits can activate PKC to modulate various targets, including T-type channels 56, 57. Interestingly, studies examining the PKC-mediated modulation of T-type channels have conflicting conclusions. For instance, PKC-mediated T-type channel inhibition was identified in reticular thalamic neurons 58. Similarly, in MN9D dopaminergic cells T-type channel inhibition induced by corticotrophin-releasing factor was prevented by PKC inhibitors 59. In contrast, stimulation of PKC either by insulin-like growth factor in dorsal root ganglion neurons or by angiotensin-II in neonatal cardiomyocytes significantly increases the current density of *I*_T_ 28, 60. Similar results have also been reported in *Xenopus* oocytes expressing recombinant T-type channels 61 but could not be reproduced in mammalian cells 56, 57, 61. Nevertheless, it is unlikely that G_βγ_-dependent PKC was involved in the NmbR-mediated T-type channel response since PKC inhibition did not affect the Nmb-induced *I*_T_ increase. Consistently, it has been shown that the addition of Nmb to Rat-1 cells transfected with the Nmb-preferring receptor activated p42 MAPK in a PKC-independent and pertussis toxin-insensitive manner 62. Our findings further suggest that AMPK-dependent PKA activity was involved in the Nmb-induced T-type channel response because 1) the AMPK inhibitor blocked Nmb-induced PKA activation and 2) pretreatment of TG neurons with either the PKA inhibitor KT-5720 or the AMPK inhibitor compound C abolished the Nmb-induced *I*_T_ response. PKA is an important effector enzyme commonly activated by cAMP. However, in perivascular adipose tissue, cAMP-independent PKA signalling has also been described 63. It has also been shown that PKA activity can be stimulated by G_βγ_ released from either G_i/o_ or G_q_ proteins, thereby regulating T-type channel activity. Consistent with our present findings, PKA has been shown to enhance *I*_T_ recorded from recombinant Cav3 channels 64, 65. Activation of PKA by serotonin type 7 receptor increases T-type channel currents in rat glomerulosa cells 66. Similar results were obtained in mouse cardiac myocytes 67. Interestingly, the opposite effect of PKA signalling has also been reported in T-type channels. For example, dopamine-mediated *I*_T_ inhibition in retinal horizontal cells and adrenaline-mediated *I*_T_ inhibition in newt olfactory receptor cells are prevented by PKA inhibitors 56. Moreover, PKA signaling was shown to inhibit Cav3.2 channels in cerebral arterial smooth muscle cells 68. The distinct modulation of T-type channel activity by PKA is likely dependent on its local microenvironment due to tissue-specific activation of endogenous PKA 69, 70 or cell-specific splice variants of T-type channels 12, 71.

It has been well established that T-type channels promote Ca^2+^ entry that disrupts the resting membrane potential and can modulate neuronal activity in the peripheral and central nervous systems, thus improving transmission efficiency 72. In the peripheral sensory neurons this low activation threshold property of T-type channels typically results in the amplification of sensory signals, increased afferent sensory transmission and, in particular, increased pain perception 73, 74. Accumulating evidence has demonstrated that inhibition of peripheral Cav3.2 T-type channels leads to significant analgesic effects in a variety of animal neuropathic pain models 19, 28, 44. In this study, we found that NmbR activation increased the excitability of mouse small TG neurons and induced mechanical hypersensitivity to chronic inflammatory pain in mice. These effects were attenuated via pharmacological blockade of PKA or Cav3.2 T-type channels or knockdown of T-type channel expression by Cav3.2-specific siRNA. Though other potential molecular targets downstream of NmbR, such as PKA-driven TRPV1 and HCN2 channels 4, involved in Nmb behavioral responses need further investigations, the NmbR-mediated stimulation of T-type channels is at least partially dependent on PKA activation in peripheral sensory neurons. These findings are consistent with previous studies that application of Nmb can induce pro-nociceptive effects 4, 5. In line with that, the administration of PKA inhibitor decreases the average number of CFA-induced nocifensive withdrawal responses to mechanical stimulation 75. By contrast, Wan and colleagues have reported normal acute thermal and chemical pain behaviors in mice lacking both NmbR and GRPR 76. One of the explanations to this discrepancy is that the systemic gene knockout could result in long-term compensatory mechanisms in the transgenic animals. Whether this is the case found in the NmbR-knockout mice requires further study. Moreover, different expression patterns (central nervous system *vs.* peripheral sensory neurons) can engender distinct or even opposing roles in regulating animal behaviors. In our study, the application of NmbR antagonist through peripheral intra TG-injection is local, highly selective, and short-term; therefore, the observed behavioral responses are less likely due to other systemic confounding effects. Chronic pain, such as inflammatory or neuropathic pain, is a common clinical symptom. Although they share some common signaling messengers, the regulation of receptors, ion channels, neurotransmitters and neuromodulators at transcriptional and translational levels may differ considerably in distinct pain models 77, 78. For example, CFA injection as an inflammatory pain model increased substance P, CGRP, and PKCγ in the spinal cord. In contrast, L5 spinal nerve ligation as a neuropathic pain model significant decreased substance P and CGRP in both sensory neurons and the spinal cord. Whereas, in a model of cancer pain, there were no detectable changes in any of these markers in either primary afferent neurons or the spinal cord 77. In the present study, we focused on inflammatory pain, and have identified Nmb as an agent that contributes to CFA-induced chronic inflammatory pain. Our present results are supported by previous studies that administration of Nmb antagonist greatly attenuates edema and nerve sensitization following stimulation of peripheral nerves with mustard oil 4. This demonstrates that Nmb contributes to neurogenic inflammation. Nonetheless, it is also important to further determine the role of Nmb in different pain models such as neuropathic pain or cancer pain models, as clinical research has shown an important role of anti-inflammatory cytokines in neuropathic and other chronic pain states in humans 79. However, we believe that this is beyond the scope of the current study, and would be investigated in our future studies.

Taken together, our results present the novel molecular circuit underlying Nmb-mediated induction of T-type channels in small TG neurons in mice. G_βγ_-coupled NmbR stimulation and AMPK/PKA signalling activation contribute to Nmb-driven neuronal hyperexcitability and are associated with pain hypersensitivity *in vivo*. Our study suggests that NmbR is a potential therapeutic target for the clinical management of pain.

## Supplementary Material

Supplementary figures.

## Abbreviations

NmbR: neuromedin B receptor
TG: trigeminal ganglion
*I*_T_: T-type Ca^2+^ channel currents
Kv: voltage-gated K^+^ channels
*I*_A_: transient outward K^+^ channel currents
I_DR_: sustained delayed-rectifier K^+^ channel currents
4-AP: 4-aminopyridine
CGRP: calcitonin gene related peptide
PKA: protein kinase A
PKC: protein kinase C
AMPK: AMP-activated protein kinase
