# Supplementary material for: Neuromedin B receptor stimulation of Cav3.2 T-type Ca2+ channels in primary sensory neurons mediates peripheral pain hypersensitivity
Source: Theranostics. 2021 Sep 9;11(19):9342–57. doi: 10.7150/thno.62255 (PMC8490515; doi:10.7150/thno.62255)
Supplement: Supplementary file 1 — Supplementary figures. [file thnov11p9342s1.pdf]

Neuromedin B receptor stimulation of Cav3.2 T-type Ca^{2+} channels in primary sensory neurons mediates peripheral pain hypersensitivity

Yuan Zhang, Zhiyuan Qian, Dongsheng Jiang, Yufang Sun, Shangshang Gao, Xinghong Jiang, Hua Wang, Jin Tao

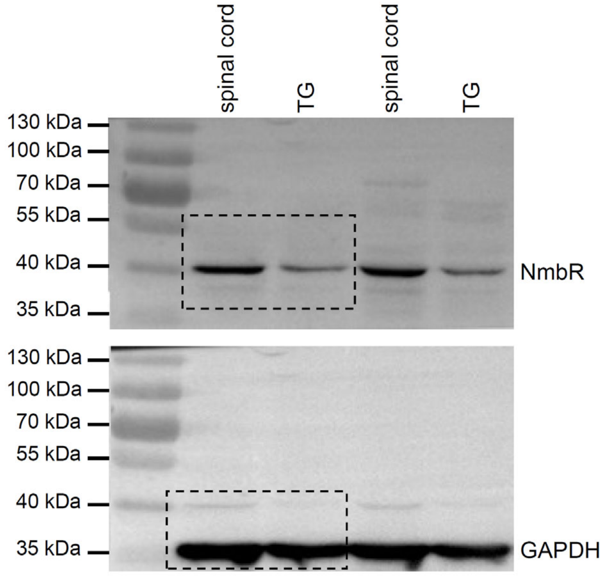

Figure S1. Abundance of NmbR in mouse TGs. Shown is the expanded image of western blot for NmbR against a loading control, GAPDH, presented in Figure 2A. Blots are representative of three experiments.

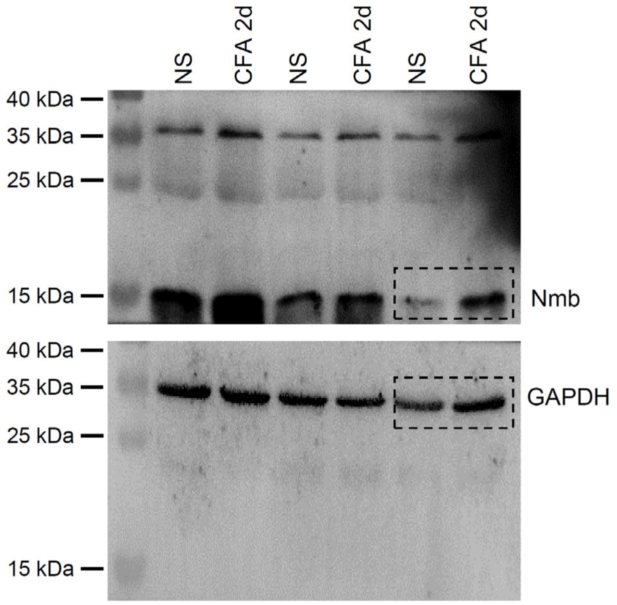

Figure S2. Increased abundance of Nmb in the local inflamed tissue induced by CFA. Shown are the full-length pictures of the blots presented in Figure 2H. Blots were representative of three separate experiments.

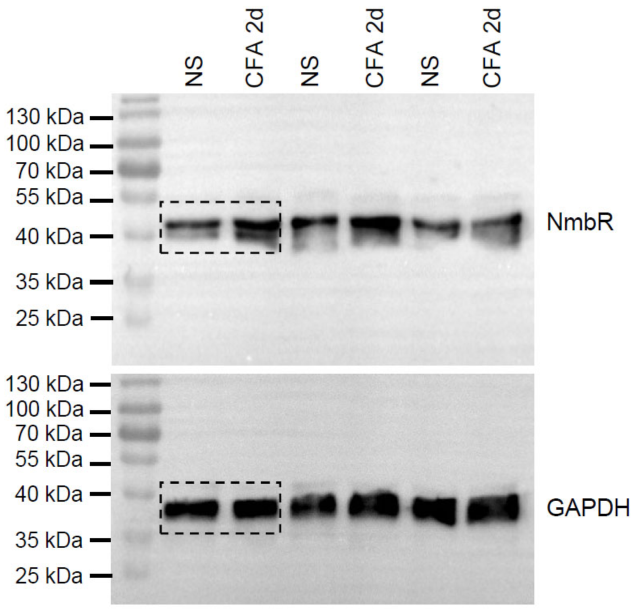

Figure S3. Increased abundance of NmbR induced by CFA. Shown are the full-length pictures of the blots presented in Figure 2I. NmbR protein expression was greatly enhanced in CFA mice when compared with age- and sex-matched controls. Blots were representative of three separate experiments.

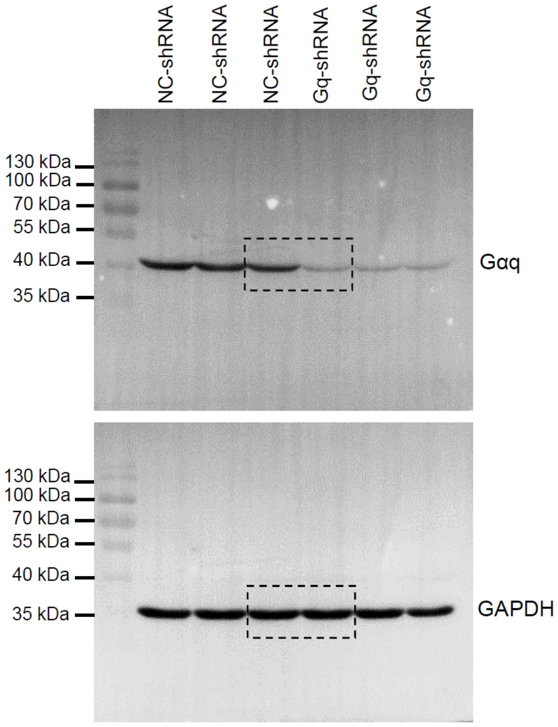

Figure S4. Knockdown of Gq in mouse TGs. Shown are the full-length pictures of the blots presented in Figure 3C. The protein abundance of Gq was measured using immunoblot analysis in negative control shRNA (NC-shRNA) and Gq shRNA-treated (Gq-shRNA) groups. Blots are representative of three experiments.

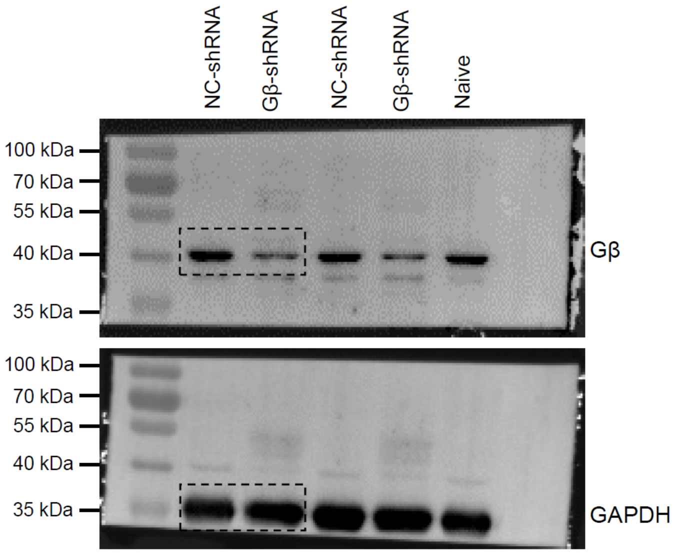

Figure S5. Knockdown of Gβ in mouse TGs. Shown are the full-length pictures of the blots presented in Figure 4C. The protein abundance of Gβ was measured using immunoblot analysis in NC-shRNA and Gβ-shRNA groups. Blots are representative of three experiments.

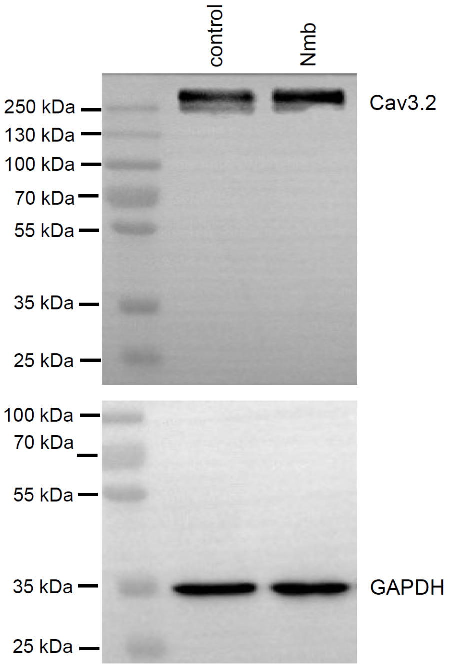

Figure S6. Change in the abundance of Cav3.2 induced by 100 nM Nmb. Shown are the full-length pictures of the blots presented in Figure 4G. Treating TG cells with 100 nM Nmb did not affect the protein expression level of Cav3.2 channels. Blots are representative of three experiments.

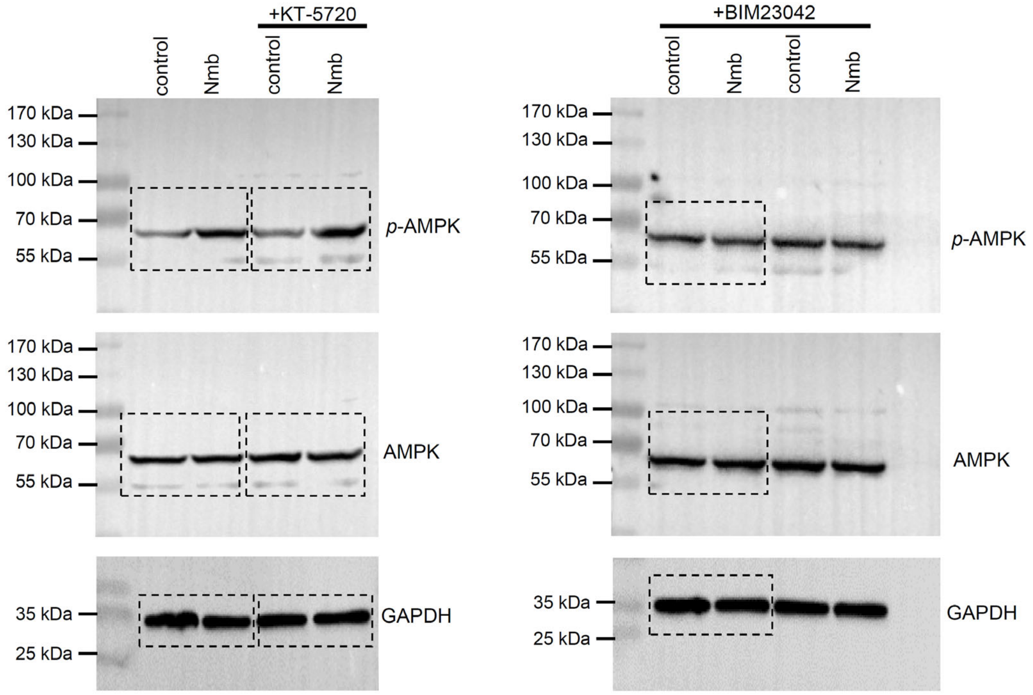

Figure S7. Change in the abundance of phospho-AMPK induced by Nmb. Shown are the full-length pictures of the blots presented in Figure 4H. Pretreating cells with BIM23042, but not 1 μM KT-5720, prevented the Nmb-induced increase in phospho-AMPK expression. GAPDH was used as the loading control. Blots are representative of three experiments.

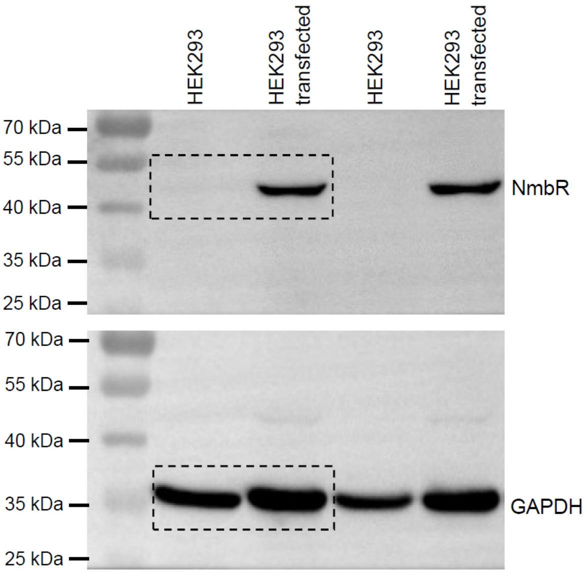

Figure S8. Abundance of NmbR in HEK293 cells. Shown are the expanded image of western blot for *NmbR*-transfected HEK293 cells against a loading control, GAPDH, presented in Figure 5A. Blots are representative of three experiments.

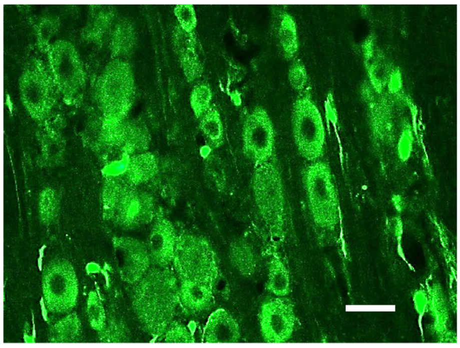

Figure S9. Representative image of 6-FAM expression with green fluorescence in an intact TG 2 d after intra-TG injection of a 5’-cholesteryl–modified Cav3.2-siRNA with 6-FAM as an expression marker. Scale bar, 50 μm.

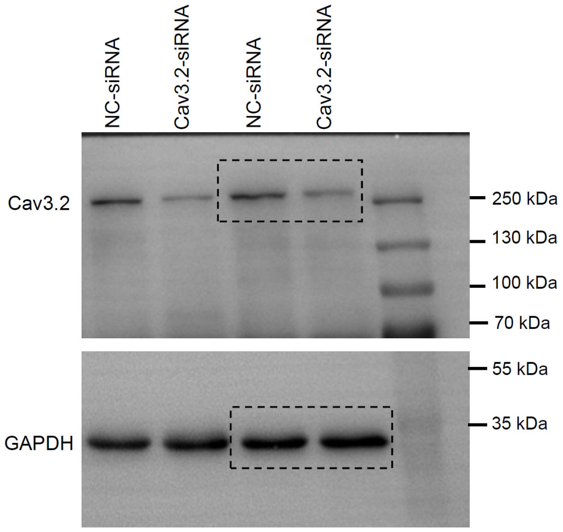

Figure S10. Knockdown of Cav3.2 in mouse TGs. Shown are the full-length pictures of the blots presented in Figure 7H. The protein abundance of Cav3.2 was measured using immunoblot analysis in negative control siRNA (NC-siRNA) and Cav3.2 siRNA-treated (Cav3.2-siRNA) groups. Blots are representative of three experiments.

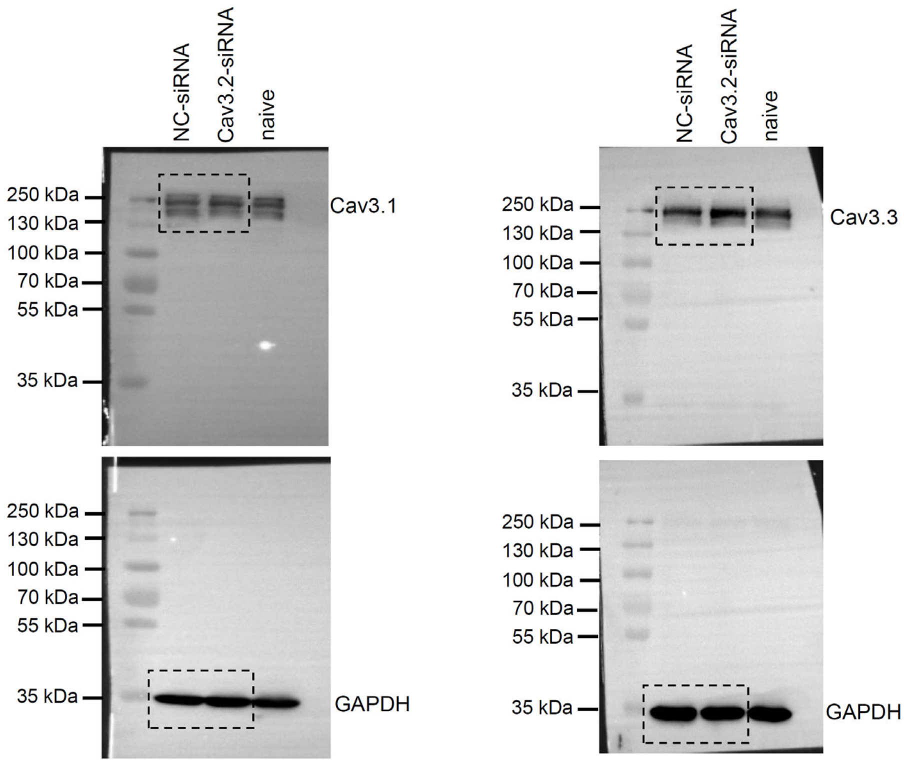

Figure S11. Effects of Cav3.2-siRNA on the protein expression level of Cav3.1 or Cav3.3 in mouse TGs. Shown are the full-length pictures of the blots presented in Figure 7I. The protein abundance of Cav3.1 or Cav3.3 was measured using immunoblot analysis in NC-siRNA and Cav3.2 siRNA-treated groups. Blots are representative of three experiments.
